# Indicators of metabolic syndrome in normotensive normoglycemic asthmatic patients

**Published:** 2020-07-13

**Authors:** Hanadi Abdelgadir Ahmed Sourg, Adil Ballal Mohammed Ahmed, Ramaze Farouke Elhakeem, Mohamed Faisal Lutfi

**Affiliations:** ^1^Faculty of Medicine, University of Khartoum, Khartoum, Sudan; ^2^Faculty of Medicine, Al Neelain University, Khartoum, Sudan; ^3^King Saud bin Abdulaziz University for Health Sciences, Riyadh, KSA; ^4^College of Medicine, Qassim University, Qassim, KSA; ^5^Nile College of Medicine Khartoum, Sudan

**Keywords:** bronchial asthma, diabetes mellitus, hypertension, insulin resistance, metabolic syndrome

## Abstract

**Background:**

Pathophysiology of hypertension and bronchial asthma (BA) shares many similarities, especially those related to the metabolic syndrome (MS).

**Aim:**

In this study, the indicators of the MS were evaluated in normoglycemic normotensive asthmatic patients to clarify if the components of the MS can still interact to increase the risk of BA, provided that blood pressure and glucose level are kept within the normal physiological ranges.

**Methods:**

Body mass index (BMI), waist circumference (WC), mean arterial blood pressure (MABP), fasting blood glucose (FBG) and fasting blood insulin (FBI) levels, the quantitative insulin sensitivity check index (QUICKI), serum lipid profile, and spirometric measurements were all compared between 120 asthmatic patients and 59 non-asthmatic subjects. Cigarette smoking, pregnancy, age below 20 years or above 40 years, diabetes mellitus and hypertension, and other chronic diseases were excluded from all studied groups.

**Results:**

Asthmatic patients demonstrated higher WC (median [25^th^-75^th^ interquartile]=88.50 [78.00-101.75], FBI [19.98 (11.12-40.14)], and triglyceride (TG) level [109.5 (76.50-134.0)]) compared with non-asthmatic subjects (81.00 [72.00-92.00], 13.78 [8.84-30.24], and 89.00 [64.25-104], *P*<0.05). QUICKI and MABP were lower in asthmatic patients (0.310 [0.283-0.338] and 86.66 [83.33-93.33]) compared with non-asthmatic subjects (0.320 [0.297-0.353] and 93.33 [83.33-93.33]), (*P*<0.05). BMI, FBG, low-density lipoprotein, high-density lipoprotein, and total cholesterol levels were comparable in the studied groups.

**Conclusions:**

The present finding gives further evidence for higher WC, FBI, TG level, and insulin resistance in normotensive, normoglycemic asthmatic patients compared to healthy controls.

**Relevance for Patients:**

The findings of this study suggested that abdominal obesity, hypertriglyceridemia, hyperinsulinemia, and insulin resistance may still be interacting and hence increase the risk of BA in normotensive, normoglycemic subjects.

## 1. Introduction

The metabolic syndrome (MS) refers to the co-existence of several recognized cardiovascular risk factors, namely, impaired glucose tolerance, obesity, dyslipidemia, and hypertension [1,2]. These components of the MS do not only increase the risk of cardiovascular diseases but also markedly worsen the pulmonary functions [3]. Previous researches demonstrated an intimate relationship between the MS and bronchial asthma (BA) [3,4], pulmonary hypertension [5,6], as well as obstructive sleep apnea [7].

Several evidences suggest epidemiological and etiological links between MS and BA [3,4]. Hyperglycemia directly induces airway smooth muscle hyperresponsiveness [8], which explains the high prevalence of BA among type 1 diabetic patients [9]. Likewise, hyperinsulinemia [10-12] and insulin resistance (IR) [13-15], which are commonly associated with type 2 diabetes mellitus, are proved to be among the risk factors for BA.

The pathophysiology of hypertension and BA shares many similarities [16]. The bronchial and vascular smooth muscles are hyperresponsive [17,18] and salt-sensitive [19,20] in asthmatic and hypertensive patients, respectively. Endocrinopathy such as IR and high renin-angiotensin activity was documented in several previous reports investigating the pathogenesis of hypertension [21,22] and BA [23,24].

The link between adiposity and BA appears to extend beyond the negative impact of obesity on pulmonary ventilation. The close relationship between BA and high body mass index (BMI) is certainly present [25-27], especially if abdominal obesity is obvious [28,29]. The association between BA and obesity can be attributed to pro-inflammatory effects of adipokines produced by the adipose tissue [30] and IR, which is common in overweight persons [13-15].

As described earlier, the pathophysiological mechanisms of hypertension and BA are not that different, especially those related to the MS. In this study, BMI, waist circumference (WC), blood pressure, fasting blood glucose (FBG) level, fasting blood insulin (FBI) level, the quantitative insulin sensitivity check index (QUICKI), and serum lipid profile were all evaluated in normoglycemic normotensive asthmatic patients. We believe that the findings of the present study will clarify if the components of MS can still interact to increase the risk of BA provided that blood pressure and glucose level are kept within the normal physiological ranges.

## 2. Materials and Methods

The ethical clearance of this study was approved by the Ethical Review Committee -Al-Neelain University Board, Khartoum, Sudan. All the studied volunteers enrolled in this study signed informed consent before being recruited for this study.

The test group consisted of 120 asthmatic patients who were recruited from Chest Clinics – Military Hospital, Khartoum, Sudan, during the period June 2016-January 2017. The control group involved 59 non-asthmatic subjects, who were co-patients, University students, and employees. The asthmatic patients were defined as self-reported, physician-diagnosed BA cases (based on clinical examination and spirometric evaluation) for at least 2 consecutive years. Cigarette smokers, pregnant women, and individuals age below 20 years or above 40 years and patients with chronic diseases such as diabetes mellitus and hypertension were all excluded from the studied groups.

Asthmatic patients were classified into controlled and uncontrolled based asthma control test (ACT) [31]. Patients with ACT <19 were considered uncontrolled [32]. Blood pressure, anthropometric, and spirometric measurements were collected using a prearranged data collection sheet. Mean arterial blood pressure (MABP) was calculated using the formula:


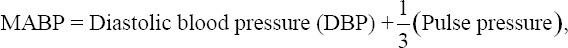


where pulse pressure is the difference between the systolic and diastolic pressure (systolic blood pressure [SBP]) and diastolic blood pressure (DBP) [33]. The examined anthropometric measurements were body weight, body height, and WC. BMI was calculated using the formula: BMI = weight (kg)/height^2^ (m^2^) [34]. The examined spirometric measurements were: The forced expiratory volume in the 1^st^ min (FEV1), the forced vital capacity (FVC), the forced expiratory volume in the 1^st^ min percent (FEV1%), and the peak expiratory flow rate (PEFR) [35].

FBG was determined by the glucose oxidase/peroxidase method (BioSystem S.A-Spain) [36]. FBI level was measured using solid-phase enzyme-linked immunosorbent assay (Immunospec Corporation-Netherlands) [37]. The QUICKI was used to estimate the degree of IR. The QUICKI index was estimated using the formula: QUICKI=1/(log FBI (mlU/ml)+log FBG (mg/dL)) [38]. Patients whose QUICKI ≤0.3 were considered IR [39].

Evaluation of lipid profile in the studied groups involved measurement of total cholesterol level using cholesterol oxidase/peroxidase enzymatic colorimetric method (BioSystem kit-BioSystem S.A-Costa Brava-Spain) [40], triglyceride (TG) level by TG glycerol phosphate oxidase/peroxidase (BioSystem S.A-Spain) [41], and high-density lipoprotein (HDL) cholesterol level by cholesterol HDL direct detergent (BioSystem S.A-Spain) [42]. Low-density lipoprotein (LDL) cholesterol level was calculated according to Friedewald *et al*. as follows: LDL = Total cholesterol – HDL – TG/5 [43].

Statistical evaluation was performed using SPSS (version 20, Chicago, SPSS Inc. USA) and OpenEpi (www.OpenEpi.com). Normal distribution of variables was examined using Shapiro-Wilk test. The normally distributed variables were described with the mean and the standard deviation (SD). Studied variables with abnormal distribution were described with the median and the 25^th^-75^th^ interquartile (Q1-Q3). Unpaired T-test was used to assess the statistical difference of the mean for normally distributed variables. Alternatively, significant statistical differences of abnormally distributed variables were assessed by comparing median (Q1-Q3) and giving the P value of the Mann-Whitney U test. The association between IR and the different studied groups was determined by Chi-square test, odds ratios (OR), and 95% confidence interval (CI). *P*<0.05 was considered significant for all statistical tests used.

## 3. Results

Distribution of age and gender were comparable in the studied groups, Table 1. In contrast, SBP, DBP, MABP, FEV1, FVC, FEV1%, and PEFR were significantly lower in asthmatic patients compared to the non-asthmatic subjects, Table 1. Although BMI was higher in asthmatic patients compared to non-asthmatic subjects, the difference in the mean did not reach statistical difference, Table 1.

**Table 1:** Characteristic of the studied groups.

	Non-asthmatic *n*=59 Mean (SD) Median (Q1-Q3) *n* (%)	Asthmatic *n*=120 Mean (SD) Median (Q1-Q3) *n* (%)	*P*
Age (Years)	28.00 (25.00-33.00)	28.00 (24.00-36.00)	0.674
Male *n* (%)	30 (50.85%)	58 (48.33%)	0.752
Weight (kg)	67.60 (58.70-79.00)	74.00 (60.00-82.95)	0.106
Height (m)	1.67 (0.08)	1.67 (0.08)	0.489
BMI (kg/m^2^)	24.13 (20.48-27.77)	26.22 (21.73-30.75)	0.060
SBP (mmHg)	120 (110.0-120.0)	110 (110.0-120.0)	0.040
DBP (mmHg)	80.00 (70.00-80.00)	70.00 (70.00-80.00)	0.003
MABP (mmHg)	93.33 (83.33-93.33)	86.66 (83.33-93.33)	0.018
FEV1 (L)	2.72 (2.30-3.69)	2.10 (1.67-2.52)	< 0.001
FVC (L)	3.50 (2.95-4.54)	2.90 (2.18-3.38)	< 0.001
FEV1%	82.99 (74.26-88.07)	75.60 (67.63-82.46)	< 0.001
PEFR (L/min)	410.0 (350.0-510.0)	300 (242.5-360.0)	< 0.001

The characteristics of the studied asthmatic patients are summarized in Table 2.

**Table 2:** Characteristic of the studied asthmatic patients.

Age of BA onset, (Years), M±SD	19.15±9.63
Duration of asthma (Years), M±SD	10.68±7.94
Controlled asthmatic patients (ACT score ≥19), *n* (%)	60 (50%)
BMI	
Underweight, *n* (%)	11 (9.2%)
Normal weight, *n* (%)	42 (35.0%)
Overweight, *n* (%)	35 (29.2%)
Obese, *n* (%)	32 (26.7%)
Anti-asthma medications	
Off-treatment asthmatic patient, *n* (%)	20 (16.7%)
Asthmatic patient on beta-agonist only, *n* (%)	45 (37.5%)
Asthmatic patient on combined beta-agonist and steroid, *n* (%)	48 (40.0%)


**Figure 1:**
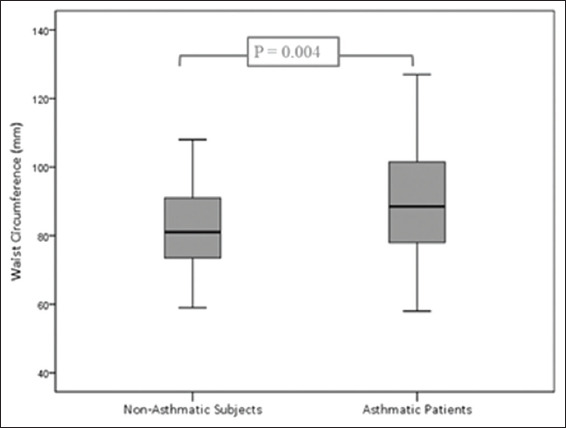
Comparison of waist circumference between asthmatic and non-asthmatic subjects.

WC was higher in asthmatic patients, Figure 1.

Insulin and TG levels were higher in asthmatic patients (compared to the non-asthmatic subjects), Table 3. Alternatively, QUICKI was lower in asthmatic patients compared with the non-asthmatic subjects. FBG, LDL, HDL, and total cholesterol levels were all comparable in the studied groups, Table 3.

**Table 3:** Comparison of biochemical indicators of MS between asthmatic and non-asthmatic subjects.

Variable	Non-asthmatic *n*=59 Mean (SD) Median (Q1-Q3)	Asthmatic *n*=120 Mean (SD) Median (Q1-Q3)	*P* value
FBG (mg/dl)	77.00 (69.00-92.00)	80.50 (72.25-96.00)	0.145
Insulin (mU/l)	13.78 (8.84-30.24)	19.98 (11.12-40.14)	0.034
QUICKI	0.320 (0.297-0.353)	0.310 (0.283-0.338)	0.024
TG (mg/dl)	89.00 (64.25-104)	109.5 (76.50-134.0)	0.006
Cholesterol (mg/dl)	200 (174.0-234.0)	198 (165.7-236.0)	0.476
LDL (mg/dl)	125.77 (49.38)	116.27 (49.80)	0.171
HDL (mg/dl)	55.00 (48.25-69.25)	57.50 (45.75-73.50)	0.982

**Figure 2:**
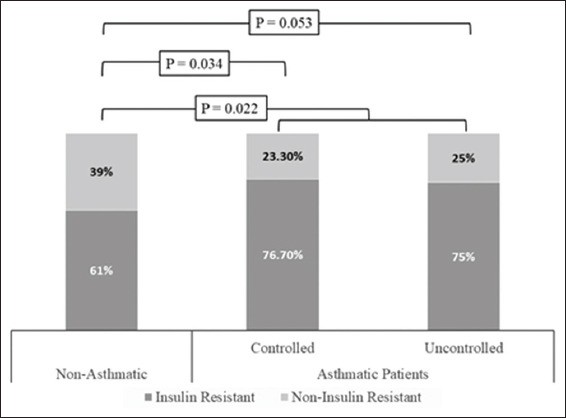
Distribution of IR among studied groups when classified according to asthma control test.

**Figure 3:**
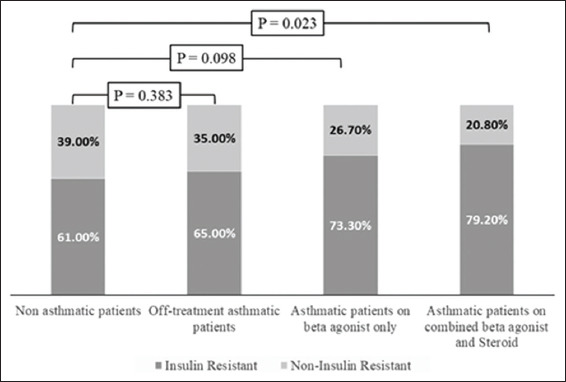
Distribution of IR among studied groups when classified according to anti-asthma medications.

IR was higher among asthmatic patients compared to the control group (OR: 2.01, 95% CI: 1.03-3.92). IR was comparably higher in controlled (OR: 2.10, 95% CI: 0.95-4.65) and uncontrolled asthmatic patients (OR: 1.92, 95% CI: 0.87-4.20) compared to non-asthmatic subjects, Figure 2. The odds of having IR increased with the increase in the intensity of asthma treatment (OR: 1.19, 95% CI: 0.41-3.42 in off-treatment asthmatic patients, OR: 1.76, 95% CI: 0.76-4.08 in asthmatic patients on beta-2 agonist only and OR: 2.43, 95% CI: 1.02-5.80 in asthmatic patients on combined beta-2 agonist and steroid therapy), Figure 3 and Table 4.

**Table 4:** Comparison between asthmatic patient with different intensity of BA treatment.

	Off treatment-asthmatic *n*=20	Asthmatic patient on beta-agonist only *n*=45	Asthmatic patient on combined beta-agonist and steroid *n*=48
Age (Years), median (Q1-Q3)	28 (22-37.3)	26 (23.5-31)	23 (21-36)
Male, *n* (%)	12 (60%)	19 (42.2%)	21 (43.8%)
BMI (kg/m^2^), median (Q1-Q3)	23.9 (20.2-28.4)	25.2 (19.9-29.5)	21.8 (21.7-27.0)
FEV1%, median (Q1-Q3)	75.9 (66.4-84.2)	72.0 (66.8-79.4)	78.6 (72.5-81.6)
PEFR (L/min), median (Q1-Q3)	373.7 (316.3-436.3)	306.5 (262.0-409.5)	253.4 (237.4-332.0)
IR, *n* (%)	13 (65%)	33 (73.3%)	38 (79.2%)

## 4. Discussion

The discussion of the present findings includes the pattern of change in the well-documented indicators of MS among the studied asthmatic patients.

### 4.1. Obesity

Although BMI was higher in the studied asthmatic patients compared to non-asthmatic subjects, the difference in the mean did not reach statistical significance. WC was higher in asthmatic patients compared to the control group. These findings are not biased by the distribution of age and gender, which were comparable among the studied groups.

Previous studies repeatedly showed a close relationship between BA and high BMI [25-27]. At the beginning of this century, 17 years or older participants of the Third National Health and Nutrition Examination Survey (NHANES III) were investigated for the possible association between BMI and the risk of BA. Results demonstrated the greatest risk of self-reported asthma among those with the highest BMI quintile [25]. NHANES III finding was further supported by a larger-scale study on Dutch adults aged 20 years or older [26]. In a separate study, an increased risk of BA was demonstrated in women who gained weight after the age of 18 years [27].

According to Luder *et al.*, the prevalence of BA in men is likely to increase if BMI <22 kg/m^2^ or ≥30 kg/m^2^ [44]. This U-shaped relationship was more apparent within the age range 18-49 years. The findings of Luder *et al*. were further supported by a Chinese study that proved the association between airway hyperresponsiveness and extremes of BMI range, both in men and women [45]. The same U-shaped relationship between BMI and airway hyperresponsiveness was also demonstrated in the results obtained from the European Community Respiratory Health Survey [46]. The high risk of BA in both extremes of BMI range might explain why the difference in BMI of studied asthmatic and non-asthmatic subjects failed to reach a statistical significance. In contrast, WC was significantly higher in the studied asthmatic patients compared to the control subjects. This finding confirms the importance of abdominal obesity as a risk factor for BA, as proved by previous reports [28,29]. Actually, large WC increases the risk of BA prevalence even among females within the physiological range of BMI [28]. According to HUNT study, abdominal obesity was still an important risk factor for the development of BA even after the adjustment for BMI [29].

### 4.2. Blood pressure

Although several studies suggested the association between BA and hypertension [47,48], other reports confirmed the presence of low blood pressure during asthma attacks, especially in pediatric wards [49-51].

It seems evident that hypertension and BA share some similarities in their pathophysiology [16]. Smooth muscle fibers of asthmatic and hypertensive patients were proved to be hyperactive [17,18] and salt sensitive [19,20]. Endocrinopathy such as IR and high renin-angiotensin activity was documented in several previous reports that investigated pathogenesis of hypertension [21,22] and BA [52,53]. Another risk factor for hypertension is stress, which is commonly associated with acute exacerbation of asthma [54].

The findings of the present study showed significantly lower systolic, diastolic, and MABP among asthmatic patients compared to the control group. This finding should be interpreted bearing in mind that hypertension was among the exclusion criteria upon the selection of the volunteers in the present study. Based on the literature review, the relatively lower readings of the blood pressures in the studied asthmatic patients may be explained by the adverse effects of beta-2 agonist used for BA treatment [49,50] as well as immunoglobulin E (IgE) -induced reaction in patients suffering from atopic BA [51].

Wisecup *et al*. investigated blood pressure in patients treated with continuous albuterol inhalation because of status asthmaticus [49]. Their results confirmed diastolic hypotension in 90% of the studied patients. In addition, the DBP correlated negatively with the doses of albuterol administered. The odds of hypotension were less among asthmatic patients who received fluid infusion before the start of albuterol nebulization. Wisecup *et al*. findings were further supported by another study that evaluated two patient cohorts: The first included patients with respiratory distress who received at least 10 mg of albuterol, and the second involved patients who had continuous albuterol nebulization because of status asthmaticus [50]. The results demonstrated significantly low DBP in 56% and 98% of the first and second cohorts, respectively. Similar to Wisecup *et al*. finding, diastolic hypotension linearly correlated with albuterol dose in both cohorts [50].

Noteworthy, it is difficult to hypothesize that adverse effects of beta-2 agonist used for BA treatment can explain as much as 10 mmHg difference in blood pressure between asthmatic and non-asthmatic, as shown earlier in the results. This marked difference in blood pressure should motivate researchers for further investigation; however, IgE-induced reaction in patients suffering from atopic BA is likely to be among the explanations. Recent studies suggested that IgE induces downregulation of Na^+^/Ca^++^ exchanger protein-1 (NCX1) expression in vascular smooth muscles [51]. NCX1 is essential in preserving a high Ca^++^ level in arterial smooth muscles during periods of vasoconstriction [55]. The role NCX1 in maintaining normal blood pressure gives a sensible explanation for asthma-induced hypotension, where IgE induces downregulation of NCX1 [51].

### 4.3. IR

Although blood glucose level was higher in the studied asthmatic patients compared to non-asthmatic subjects, the difference in the mean did not reach statistical difference. This finding is expected since diabetes mellitus was among the exclusion criteria upon selection of the volunteers of the present study. In spite of the statistically comparable blood glucose levels in the studied groups, the insulin level was significantly higher in the asthmatic patients compared to the control subjects, which suggested increased susceptibility of IR in the first group. This hypothesis is further supported by the significantly lower values of QUICKI in the studied asthmatic patients compared to the non-asthmatic subjects. According to the present results, the odds of having IR are doubled in asthmatic patients compared to the control subjects, regardless of the degree of asthma control. Alternatively, the odds of having IR increased with the increase in the intensity of BA treatment received.

In a previous Sudanese study on the hyperglycemic effect of BA, Lutfi and Sukkar compared the readings of random blood glucose (RBG) levels of 41 apparently healthy subjects to a group of 72 patients with BA, after being classified according to ACT and anti-asthma treatments [56]. Similar to the present study, both the test and the control groups were non-diabetic and non-hypertensive. The mean of RBG in the control group was significantly lower compared to those of the controlled as well as the uncontrolled asthmatic patients. In addition, RBG of non-asthmatic patients was statistically decreased compared with off-treatment asthmatic patients, patients treated with beta-agonists only, and patients treated by combined therapy [56].

The association between IR and BA is supported by a recent cross-sectional study performed on adolescents aged 12-17 years from National Health and Nutrition Examination Survey [13]. Adjusted regression performed on the data gained from the survey demonstrated a negative association between IR and both FEV1 and FVC [13]. Another study conducted in Seoul National University Hospital Gangnam confirmed the association between IR and bronchial hyperreactivity, which is the most distinguishing feature of BA [14,15]. Improvement in pulmonary function following the treatment of non-diabetic asthmatic patients was associated with a parallel improvement in insulin sensitivity [57]. A study involving Canadian children, aged 7-8 years, suggested that maternal diabetes mellitus may be involved in perinatal programming of childhood asthma [58].

Previous efforts toward the use of inhaled insulin formulations for the treatment of diabetes mellitus gave insights on the possible direct effects of insulin on the respiratory airways [59]. Clinical trials on the use of inhaled insulin in diabetic patients confirmed the significant decrease in FEV1 of the targeted patients [60]. Although the mechanism of FEV1 reduction was not obvious, it did not progress at least over 2 years of the follow-up and was reversible following withdrawal of the treatment [12]. Other studies on possible effects of insulin on the immune system demonstrated the shift of the T lymphocytes toward predominance of type-2 (Th-2) over type-1 T-helper cells (Th-1) [10], which is a known key etiological event that ultimately induces development of BA [11]. Moreover, insulin was proved to increase mast cell life span and degranulation and consequently enhances airways hyperreactivity [61].

In spite of the aforementioned studies on the pro-inflammatory effects of insulin and the possible contribution of hyperinsulinemia in the pathogenesis of BA, other reports demonstrated anti-inflammatory effect of insulin [62-65]. Insulin exerts a vasodilatory effect through attenuation of norepinephrine-induced venoconstriction [62], as well as the enhancement of nitric oxide release from endothelium [63]. Vasodilatation decreases margination and diapedesis of leukocytes and subsequent inflammation [64]. In addition, previous researches repeatedly reported inhibitory effects of insulin on a wide spectrum of inflammatory mediators [64,65].

Although the researches that uncovered the pro-and anti-inflammatory effects of insulin appeared contradictory, these reports do not oppose the findings of the present study. Hyperinsulinemia and IR were both obvious in the studied asthmatic patients compared to the control group. In the studied asthmatic patients, the pro-inflammatory effects secondary to hyperinsulinemia seemed to be augmented by the lack of anti-inflammatory effects due to IR. However, further researches are desirable to clarify this hypothesis and to postulate the possible synergistic effects of hyperinsulinemia and IR on the pathogenesis of BA.

### 4.4. Lipid profile

The present results showed significantly higher TG levels in asthmatic patients compared to the control subjects. In contrast, LDL, HDL, and total cholesterol levels were comparable in the studied groups. These findings are comparable with van Zelst *et al.*, who demonstrated increased serum TG levels, but comparable LDL and total cholesterol levels in asthma patients with high BMI compared to healthy controls in the same BMI-range [66]. Analysis of Korea National Health and Nutrition Examination Survey data revealed no significant difference of TG, LDL, HDL, and total cholesterol levels between of asthmatic patients aged 11 and 18 years and the control group [67]. Alternatively, different findings were displayed in another study investigating 10-15-year-old children from Northern Taiwan [68]. The total and LDL cholesterol levels increased progressively in Taiwanese children being highest in obese asthmatics followed by non-obese asthmatics, the obese controls, and lastly, non-obese controls in descending order. Ramaraju et al. assessed the total cholesterol levels in 40 adult asthmatic patients and a similar number of control subjects [69]. Their finding revealed a significant association between total cholesterol and BA, which was independent of age, gender, BMI, and socioeconomic status. However, there was no association between total cholesterol and disease characteristics such as duration of BA, intensity of treatment, and number of emergency hospital visits [69]. A recent meta-analysis study on the association between lipid profile and the prevalence of asthma concluded that levels of total LDL cholesterol are higher in patients with asthma than those of non-asthmatic subjects [70].

Although the association between dyslipidemia and BA was reproduced by several previous reports, the exact mechanism for this association is unclear and remained for further investigations. Dyslipidemia is a known indicator of IR, which is common among patients with BA, as described earlier [13-15,57,58]. Hypercholesterolemia has a pro-inflammatory effect, persuading the secretion of inflammatory mediators [71] and enhancing expression of endothelial cellular adhesion molecules [72]. Moreover, dyslipidemia is a known risk factor for eosinophilic inflammation, which can exacerbate BA by enhancing mucus secretion and bronchial smooth muscle hyperresponsiveness [73].

Although the present results showed significantly higher TG levels in the asthmatic patients compared to the control subjects, LDL, HDL, and total cholesterol levels were comparable in the studied groups. This finding should be interpreted bearing in mind that major associations with hypercholesterolemia such as hypertension and diabetes mellitus were among the exclusion criteria upon the selection of the volunteers in the present study. Such exclusion criteria might have acted as cofounders that precluded the marked differences in cholesterol levels between the study groups.

### 4.5. Study limitations

The asthmatic patients examined in the present study were limited to otherwise healthy, 20-40 years of age, non-smokers so as to control for possible risk factors of hypertension and IR. However, the modulatory effects of smoking, age, and comorbidities related to obesity should be considered in further studies intended to evaluate indicators of MS in normoglycemic normotensive asthmatic patients.

The present study did not assess the presence of atopy among the studied groups. Evaluation of IgE levels and skin-prick test, if considered, could have clarified whether the indicators of MS are linked to atopy. Likewise, the study did not measure the level of important cytokines and other chemical mediators involved in the pathogenesis of BA, Th-2/Th-1 ratio, differential count of eosinophils, and other inflammatory cells. Assessment of these markers in future researches will definitively give better insights about the possible links between MS, IR, and BA. Follow-up of the currently examined indicators of MS in the same studied groups in a prospective cohort will be beneficial to identify the risk of diabetes mellitus and hypertension in the asthmatic patients involved in this study.

## 5. Conclusions

The current finding suggests that abdominal obesity, hypertriglyceridemia, hyperinsulinemia, and IR may still interact in normotensive, normoglycemic subjects to increase the risk of BA. In contrast, BMI and serum cholesterol level failed to show the usual trend of MS in the studied asthmatic patients. Although the blood pressure was within the normal physiological range, it was relatively lower in asthmatic patients as compared to the control group. This is probably because of the adverse effects of beta-2 agonist used for BA treatment or IgE-induced reaction in atopic asthma patients. The implications of this study remain open for further investigations to explain their molecular and cellular basis.

### Ethics Approval and Consent to Participate

The study received ethical approval from the Ethical Review Committee -Al-Neelain University Board, Khartoum, Sudan. All studied subjects signed informed consent before being enrolled in the study.

### Consent for Publication

Not applicable.

### Availability of Data and Materials

The data supporting the present findings are contained within the manuscript.

### Competing Interests

None to declare.

### Funding

None to declare.

### Authors’ contributions

MFL designed the study. HAAS and ABMA carried out experimental protocols. MFL and HAAS analyzed the data. MFL and RFE prepared the manuscript draft. All authors read and approved the final manuscript.
